# KBNet: A Language and Vision Fusion Multi-Modal Framework for Rice Disease Segmentation

**DOI:** 10.3390/plants14162465

**Published:** 2025-08-08

**Authors:** Xiaoyangdi Yan, Honglin Zhou, Jiangzhang Zhu, Mingfang He, Tianrui Zhao, Xiaobo Tan, Jiangquan Zeng

**Affiliations:** 1 College of Electronic Information & Physics, Central South University of Forestry and Technology, Changsha 410004, China; 20223793@csuft.edu.cn (X.Y.); 20201387@csuft.edu.cn (H.Z.); 20223799@csuft.edu.cn (T.Z.); 20241200783@csuft.edu.cn (X.T.); 2 College of Computer & Mathematics, Central South University of Forestry and Technology, Changsha 410004, China; 20235366@csuft.edu.cn

**Keywords:** rice disease, segmentation, multi-modal, feature extraction

## Abstract

High-quality disease segmentation plays a crucial role in the precise identification of rice diseases. Although the existing deep learning methods can identify the disease on rice leaves to a certain extent, these methods often face challenges in dealing with multi-scale disease spots and irregularly growing disease spots. In order to solve the challenges of rice leaf disease segmentation, we propose KBNet, a novel multi-modal framework integrating language and visual features for rice disease segmentation, leveraging the complementary strengths of CNN and Transformer architectures. Firstly, we propose the Kalman Filter Enhanced Kolmogorov–Arnold Networks (KF-KAN) module, which combines the modeling ability of KANs for nonlinear features and the dynamic update mechanism of the Kalman filter to achieve accurate extraction and fusion of multi-scale lesion information. Secondly, we introduce the Boundary-Constrained Physical-Information Neural Network (BC-PINN) module, which embeds the physical priors, such as the growth law of the lesion, into the loss function to strengthen the modeling of irregular lesions. At the same time, through the boundary punishment mechanism, the accuracy of edge segmentation is further improved and the overall segmentation effect is optimized. The experimental results show that the KBNet framework demonstrates solid performance in handling complex and diverse rice disease segmentation tasks and provides key technical support for disease identification, prevention, and control in intelligent agriculture. This method has good popularization value and broad application potential in agricultural intelligent monitoring and management.

## 1. Introduction

Rice is one of the most vital crops globally, forming the dietary foundation for much of humanity while ensuring food security on a massive scale [1]. However, during the growth process, rice is vulnerable to various pests and diseases, which seriously threaten its yield and quality, and place huge pressure on agricultural production [2]. According to research, pests and diseases significantly reduce global rice production each year, with estimated losses reaching tens of millions of tons [3]. These reductions lead to substantial economic damage and pose serious challenges to global food security. Traditional control methods usually rely on farmers’ field observation or agricultural experts’ field diagnosis, but these methods have problems such as cumbersome operation, low efficiency, and subjective factors affecting accuracy [4]. Therefore, the accurate identification and regional division of rice pests and diseases with the help of image segmentation technology has become one of the key paths to promote the development of agricultural intelligence. Image segmentation can not only realize the pixel-level positioning of the lesion area but also provide basic data support for subsequent disease identification, statistical analysis, and prevention decisions [5].

The agricultural sector has recently seen a growing implementation of machine vision, driven by innovations in image processing [6]. The evolution of deep learning and other AI methods has greatly strengthened the functionality of image processing, offering substantial support for disease recognition. Among them, image segmentation technology, as a key link, plays a vital role in the accurate identification of rice diseases. In the development of image segmentation technology, models such as UNet [7], DeepLab [8], and Mask R-CNN [9] have been widely used in fine image analysis tasks. They enable pixel-wise segmentation, which helps in clearly distinguishing the target zone from the surrounding background [10]. The CNN-based [11] deep neural network architecture, with its encoding-decoding structure, demonstrates considerable performance benefits in image segmentation tasks. Through multi-scale feature extraction and end-to-end training strategies, these models can capture the complex spatial structure information in the image and achieve more accurate region segmentation and semantic understanding [12]. For example, Zhang et al. [13] proposed an improved U-Net network structure, MU-Net, which improved the transmission efficiency of characteristic information between the contraction path and expansion path by introducing a residual block and residual path. However, when MU-Net is used to process leaf images with blurred edges or disease spots close to the background color, there may still be a problem that the segmentation boundary is not fine enough, affecting the accuracy of subsequent disease recognition. Lu et al. [14] proposed a white blood cell segmentation method WBC-Net, based on UNet++ [15] and ResNet. By designing a context-aware feature encoder and combining convolution and residual blocks, this method enhances the segmentation accuracy of white blood cell images by extracting multi-scale features. However, the training effect of WBC-Net under the condition of small samples still depends on a large amount of data and high-quality annotation, which limits its popularization and application in low-resource scenarios. The progress made in these areas showcases the effectiveness of different methods for image segmentation, as well as their respective strengths and weaknesses in various application contexts. With the continuous progress of agricultural technology, single-mode data has been unable to fully describe the growth state of crops in a complex environment [16]. The data from different modes provide crop information from multiple perspectives, which makes it possible for more precise disease identification. In the past few years, the advancement of deep learning techniques and multi-modal sensing technologies has led to increased focus on integrating image, text, sensor, and other data types, fostering research in multi-modal segmentation. The multi-modal method realizes information complementarity and fusion by modeling the relationship between multi-source data, and shows stronger recognition and expression ability in complex scenes [17]. In the context of rice disease segmentation, multi-modal techniques merge sensor data to improve the segmentation’s accuracy and resilience. Hu et al. [18] proposed a language vision fusion tomato disease segmentation framework, LVF, which effectively enhanced the disease segmentation accuracy in a complex agricultural environment by designing a differential fusion network (RIFN), feature enhancement network (GCM), and cosine screening network (COA). Although the LVF framework significantly improves the segmentation accuracy of tomato disease through multimodal fusion, its overall network structure is complex and requires high computing resources and training time. Li et al. [19] proposed a multimodal tomato leaf disease detection model, PDC-VLD. By integrating global and local features, introducing a denoising mechanism, and optimizing learning efficiency, the accuracy and generalization ability of disease detection are improved. Although the PDC-VLD model has achieved significant performance improvement in the detection of tomato leaf diseases, its detection effect is still vulnerable to the interference of complex backgrounds, and it is difficult to maintain stable recognition performance under the condition of small samples in the real environment.

**Figure 1:**
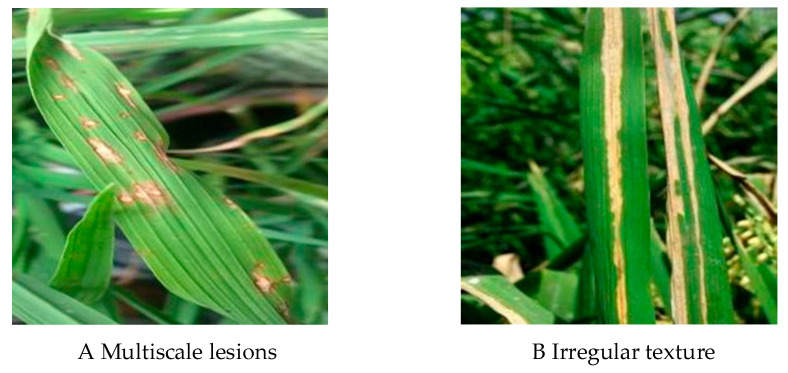
Two challenges in rice pest and disease segmentation.

Despite the significant progress made in segmentation through these studies, there remain certain drawbacks and two key challenges:

(a) In practical applications, rice disease segmentation often faces the problem of multi-scale disease spots that are difficult to deal with. Due to the diversity of lesion shape and size, the same disease can manifest different characteristics at various stages of plant growth, which makes it difficult to accurately extract the disease area. As shown in Figure 1A.
(b) During the growth process of rice disease spots, they often show the characteristics of irregular diffusion and usually have complex texture and shape. This irregularity makes it difficult to accurately capture the lesion boundary, which is prone to identification confusion or misjudgment. As shown in Figure 1B.

In order to solve the problem that multi-scale disease spots are difficult to handle, Tuncer et al. [20] proposed a multi-level fusion feature generation module (MF-GM), which aims to improve the feature extraction ability of driving fatigue detection in EEG signals [21]. This technique merges binary pattern (BP) and statistical features, employing one-dimensional discrete wavelet transform (1D-DWT) [22] for multi-level decomposition to capture low, medium, and high-level features. This module improves the efficiency of feature generation by reducing the time complexity. However, this method may still be affected by the signal quality when processing EEG signals in a high-noise environment, which leads to a decrease in the accuracy of feature extraction. Xu et al. [23] proposed a selective feature extraction module (SFE) to improve the ability of the ultrasound image segmentation model to extract lesion edge details and overall structural features. By incorporating a detail extraction phase and a structure extraction phase, this method effectively captures edge details and morphological characteristics of lesions, improving the network’s ability to process fuzzy or multi-scale lesions. Although the SFE module can effectively capture the details and overall morphological characteristics of the lesion area, its multi-stage design may increase the computational overhead, which will affect the training efficiency of the model. Therefore, it is necessary to propose a more efficient method, which can still maintain high segmentation accuracy in the environment of multi-scale disease spots and noise interference. By introducing more refined feature extraction and noise suppression mechanisms, the adaptability of the model to complex lesions is enhanced.

In order to solve the problem of irregular growth and complex texture of disease spots, Borse et al. [24] proposed the InverseForm loss function, which can predict the degree of parametric spatial transformation between the boundary and the real boundary by introducing the inverse transformation network. The former focuses on capturing boundary translation, rotation, and scale changes and makes up for the lack of sensitivity of traditional loss to local spatial error. This approach effectively addresses the issue of irregular lesion segmentation, significantly enhancing the model’s accuracy in boundary regions, without adding complexity to the reasoning phase. However, the InverseForm loss function relies on accurate boundary labeling, and its performance may degrade in the face of boundary labeling errors or weak boundaries. Bougourzi et al. [25] proposed a multi-class boundary-aware cross-entropy loss function (MBA-CE). Aiming at the problem of the irregular boundary of the infection area in the CT image, this method designs a boundary perception weight item [26] to enhance the discrimination ability of boundary pixels. In the process of feature monitoring, MBA-CE further strengthened the recognition ability of the model for complex texture and fine-grained structure and significantly improved the segmentation accuracy of multiple pneumonia infection regions. Although MBA-CE enhances the perception of boundary, the boundary weight mechanism introduced by MBA-CE may cause misleading gradients in the non-boundary region, thus affecting the stability and generalization ability of the overall model. Therefore, it is necessary to propose a new method to optimize the boundary box prediction by introducing physical constraints so as to accurately locate the region of the disease spot and improve the segmentation accuracy of the disease spot. And it can reduce the phenomenon of false detection and missed detection while maintaining efficient calculation.

Although prior methodologies have demonstrated improvements in associated fields, problems such as multi-scale lesions are difficult to deal with, and irregular lesion growth still poses challenges to the performance of the model. To solve these problems, this paper mainly makes the following contributions:

1. In order to improve the recognition ability of the rice disease segmentation model for complex disease spots, we construct an image dataset covering three common rice diseases: Bacterial Blight, Blast, and Tungro. All image samples are accurately labeled pixel by pixel using the Labelme tool to generate the corresponding segmentation mask to ascertain the correctness of the lesion zone information.
2. To resolve the aforementioned challenges, we propose an accurate rice disease segmentation model using KBNet, which integrates two innovative improvements to improve the performance and stability of the model. The design is as follows:
  (a) We propose the Kalman Filter Enhanced Kolmogorov–Arnold Network module (KF-KAN), which can efficiently capture the nonlinear features of different scales through KANs and dynamically update and fuse the multi-scale information combined with the Kalman filter to realize the accurate feature extraction of complex disease spots. By adaptively adjusting the scale and details, KF-KAN contributes to a considerable increase in both the accuracy and stability of the model when handling multi-scale lesions, thus refining the overall efficiency of the segmentation task.
  (b) We propose the Boundary-Constrained Physical-Information Neural Network module (BC-PINN), which combines the prior knowledge of physical laws with the powerful modeling ability of a neural network. By embedding the physical information, such as the growth law of the lesion, into the loss function, BC-PINN can effectively constrain the prediction of irregularly growing lesions. In addition, the BC-PINN module further constrains the spatial position of the prediction result by calculating the penalty of the predicted mask and image boundary and can provide more accurate segmentation results.

## 2. Materials and Methods

### 2.1. Data Acquisition

The segmentation dataset of rice diseases is the basis of this study. We selected three typical types of rice leaf disease: Blast, Bacterial Blight, and Tungro to verify the characteristics of rice leaf disease spots under different disease conditions. Bacterial blight disease usually forms watery, yellow-brown spots on the leaves, with fuzzy edges, and easily expands along the veins. Blast disease often causes oval or spindle grayish-brown spots on the leaves, dark brown on the edges, gray in the center, and sometimes accompanied by small black spots. Tungro disease showed obvious symptoms such as chlorosis and yellowing of leaves, curling, and dwarfing of plants. We collected a total of 1550 rice leaf images from PlantVillage on the Kaggle [27] platform, among which the numbers of images for Blast, Bacterial Blight, and Tungro are 522, 518, and 510, respectively. The image environment is complex, including strong light, walls, soil, bags, weeds, and other elements. These background changes provide challenges closer to real application scenarios for disease segmentation. In the process of image screening, we carried out strict quality control on the data and deleted some bad samples, such as highly blurred images, images with heavily occluded lesion areas, etc., to ensure data clarity and labeling accuracy.

### 2.2. Data Processing

**Figure 2:**
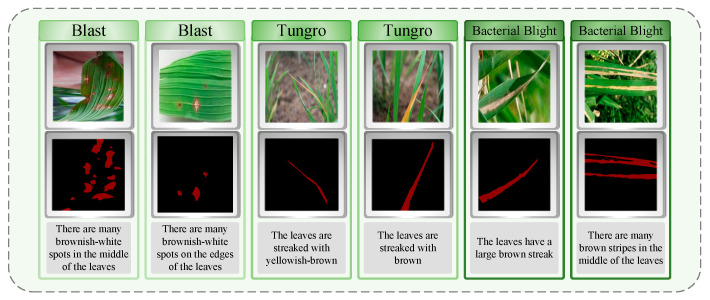
Overview of the dataset contents: labels and text descriptions of the three rice diseases.

We divided the collected images into training sets, verification sets, and test sets according to the ratio of 4:2:1. On this basis, in order to facilitate subsequent training, we use Labelme software (The version used in this study is Labelme 5.5.0.) for data annotation. Specifically, we accurately classify the lesion regions in each image and accurately outline all pixels of the lesion region. All tag masks are named after the disease type, and the corresponding JSON file is finally generated. In the process of data annotation, we received the assistance of experts from the Changsha Academy of Agricultural Sciences, Hunan Province, China, and provided a detailed text description for each rice leaf image. The content of the annotation includes key information such as the location, shape, and color of the lesion area, ensuring that the marking of each lesion area is very accurate. As shown in Figure 2, the label of the rice disease image is displayed. In order to avoid over-fitting and speed up the training, we preprocessed all the images, adjusted them to a uniform size of 224 × 224 pixels, and stored the processed images directly in the labelcol label folder as the input of the model. As the deep neural network model requires a substantial amount of varied data to identify meaningful features, we implemented data enhancement on the original dataset. Specific augmentation strategies, including geometric operations like flipping images horizontally and vertically, were used to boost data diversity and enhance the model’s generalization and robustness in varying contexts.

### 2.3. KBNet

**Figure 3:**
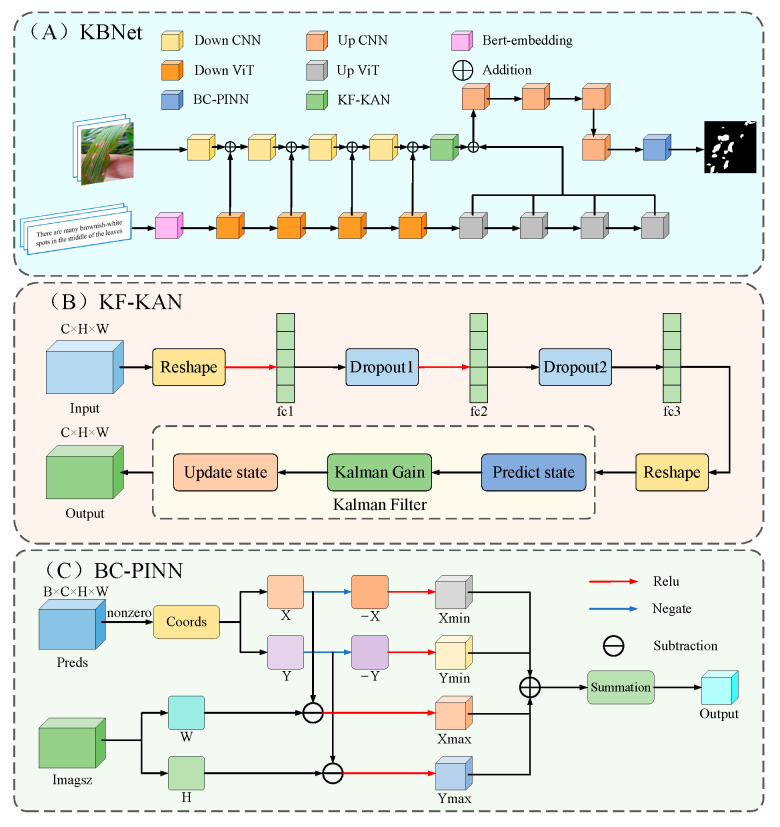
The structure of KBNet.

This study presents a rice disease segmentation approach, KBNet, which is based on LViT [28]. The architecture of the network model is illustrated in Figure 3A. In KBNet, the input image feature vector and word vector are first fused by the LViT model. The specific process is as follows: the image feature vector is first sent to the CNN network for preliminary processing. In this process, the image features are embedded through Bert, and then the input image is convoluted after the under-sampling operation. Next, the word vector and image feature matrix will be cross-fused at different scales, and these cross-nested vectors will eventually be integrated into the subsampling part of the main CNN. After several nested processing steps, a semantic image nested feature layer is finally generated. In the feature extraction process after semantic image nested feature generation, we propose the KF-KAN module. KF-KAN can accurately extract the features of complex lesion regions, especially in multi-scale disease regions. In the aspect of loss function design, we propose the BC-PINN module. BC-PINN can effectively constrain the prediction of irregular growth lesions and generate reliable monitoring signals using physical information. The module is applied before the output results, and the purpose is to optimize the features more finely through self-supervised learning.

#### 2.3.1. Kalman Filter Enhanced Kolmogorov–Arnold Networks

The KF-KAN module is a feature extraction module that combines Kolmogorov–Arnold Networks (KANs) [29] and a Kalman filter [30]. It aims to improve the accurate identification of the lesion region through dynamic updating and fusion of multi-scale features. With their powerful nonlinear mapping ability and efficient feature representation, KANs have shown excellent performance in multi-modal tasks. The KF-KAN module further introduces the Kalman filtering mechanism to enhance the processing ability of multi-scale lesion features. The Kalman filter is mainly used to dynamically update the feature information and improve the long-term tracking and fusion ability of the model through the prediction and update mechanism. One of the core components of the module is the adaptive residual structure, which combines the full connection layer and the adaptive weight allocation mechanism. Through this design, KF-KAN can dynamically adjust the weight of each layer of features according to the changes in the feature map, strengthen the extraction of key information, and reduce the interference of irrelevant features. In addition, in order to further enhance the stability of the network and avoid overfitting, the KF-KAN module uses a Dropout layer to bolster the model’s generalization capacity by randomly dropping some neurons. In the process of multi-scale lesion processing, KF-KAN effectively captures the feature changes in different scales and improves the performance of the model by integrating the nonlinear feature representation of KANs and the dynamic update of the Kalman filter. Figure 3B presents the structure of the KF-KAN module.

In the KF-KAN module, the input is a feature map with size (B,D), where B is the batch size and D is the dimension of the input feature. The module uses multi-layer full connection layer (including a Dropout layer, an activation function, and a residual connection) to gradually enhance the ability of feature representation; especially when dealing with multi-scale lesions, it can more effectively extract features at different scales. First, the input characteristic graph is mapped from the input dimension D to the hidden layer dimension H through the first layer, full connection operation. This process can be represented by Formula (1), where $W_{1}$ is the weight matrix of the first layer full connection layer, and $b_{1}$ is the offset term. The ReLU activation function introduces nonlinear characteristics to enhance the expression ability of the model. Then, it is processed by a Dropout layer to avoid over-fitting when dealing with complex scales. This operation can be represented by Formula (2):

(1) $$x_{1}=ReLU(W_{1}x+b_{1})$$

(2) $$x_{1}^{\prime }=Dropout(x_{1})$$

The features processed by the Dropout layer are sent to the full connection layer of the second layer, and the dimension is maintained as the hidden layer dimension H, further enhancing the multi-scale feature expression. The second layer output is processed by the ReLU activation function and Dropout to ensure that features can be efficiently captured when processing multiple scales. The formula is as follows:

(3) $$x_{2}=ReLU(W_{2}x_{1}^{\prime }+b_{2})$$

(4) $$x_{2}^{\prime }=Dropout(x_{2})$$

The features processed in the first two layers finally enter the third full connection layer, and the dimension is mapped from the hidden layer dimension H to the output dimension O to generate the final multi-scale feature representation. The third layer no longer uses the activation function to directly generate the output characteristics:

(5) $$x_{3}=W_{3}x_{2}^{\prime }+b_{3}.$$

In order to enhance the information transmission between different scale features, the KF-KAN module adds the input features and the final output features through the residual connection mechanism to form the fusion output of multi-scale information. This operation can be represented by Formula (6):

(6) $$\hat{x}=x+x_{3}.$$

Through residual connection, the module can realize cross-layer information transmission, ensuring that multi-scale features, ranging from low to high levels, are effectively utilized, preventing the loss of critical low-level information during deep feature extraction.

Then, the KF-KAN module inputs the output into the Kalman filter as a preliminary state estimation. The Kalman filter follows a two-step procedure: prediction and update. First, the Kalman filter predicts according to the current state and the state transition matrix F, and the corresponding formula is presented below:

(7) $${\hat{x}}_{k|k-1}=F\cdot x_{k-1}.$$

where F is the state transition matrix and $x_{k-1}$ is the state estimation at the previous time.

Next, the Kalman filter updates the state according to the actual observation value $Z_{k}$, calculates the Kalman gain K and updates the state estimation as follows:

(8) $$K=P_{k|k-1}\cdot H^{T}\cdot {\left(H\cdot P_{k|k-1}\cdot H^{T}+R\right)}^{-1}$$

(9) $$x_{k|k}=x_{k|k-1}+K\cdot (z_{k}-H\cdot x_{k|k-1})$$

where $P_{k|k-1}$ is the predicted state covariance matrix, H is the observation matrix, and R is the observation noise covariance matrix.

Finally, the Kalman filter renews the state covariance matrix as follows:

(10) $$P_{k|k}=(I-K\cdot H)\cdot P_{k|k-1}$$

where I is the identity matrix.

In this study, the Kalman filter was directly applied to neural network features without relying on state space modeling. In traditional Kalman filtering applications, the core of state space modeling is to clarify the system state transition and observation model. In the KF-KAN module, due to the powerful nonlinear mapping ability and efficient feature representation of KANs, the neural network extracts features and directly provides them to the Kalman filter as state estimation, and then uses the prediction and update mechanism of the Kalman filter to dynamically adjust and optimize the features. In order to adapt to the input of static image features, we use a fixed state transition matrix F, observation matrix H, and noise covariance matrices Q and R. These matrices are usually initialized as identity matrices or diagonal matrices with slight perturbations. Specifically, F and H are set as identity matrices to keep the state and observation space consistent, while Q and R are set as small positive definite diagonal matrices for modeling, estimating, and observing uncertainties in the process. This setting does not rely on the evolution of the time dimension but independently predicts and updates the features output by KF-KAN on each input, essentially achieving adaptive optimization of features in static input scenarios. Because KANs have already completed complex feature extraction at the feature level, they avoid the traditional state space modeling steps and directly embed the dynamic updating ability of Kalman filtering into the feature extraction process of neural networks, thereby achieving efficient fusion and adaptive adjustment of feature information.

The KF-KAN module successfully solves the challenge of multi-scale lesion feature extraction by combining the dynamic feature update mechanism of KANs and the Kalman filter. Through the fusion of nonlinear feature representation and the Kalman filter, KF-KAN captures lesion features at different scales with precision, while boosting the model’s stability and robustness through the use of adaptive residual structures and a Dropout layer. Compared with traditional methods, the KF-KAN module not only improves the ability to capture multi-scale information but also effectively avoids the interference between features and ensures the accurate identification of disease spots in complex environments. The ‘Effectiveness of the KF-KAN’ section covers experimental studies on KF-KAN and compares it with other feature extraction modules.

#### 2.3.2. Boundary-Constrained Physical-Information Neural Network

In order to effectively deal with the problem of irregular growth and complex texture of disease spots, this paper proposes the BC-PINN module, especially for the task of disease spot segmentation in rice disease images. BC-PINN can guide the neural network to segment more accurately by embedding physical information, such as the growth law of the lesion, into the loss function. The core advantage of the BC-PINN module is that it combines the prior knowledge of physical laws with the modeling ability of a neural network. In addition, the BC-PINN module constrains the spatial position of the prediction result by calculating the penalty of the predicted mask and image boundary. The loss function in the module includes the traditional Dice loss and cross entropy loss (BCE) and combines the penalty term based on physical information. When the prediction of the lesion exceeds the image boundary, the loss function will punish it so as to prevent the wrong expansion of the prediction result. Moreover, the module adaptively adjusts the weight of physical information to ensure that the physical prior can meet the training requirements of the model in different scenarios. The BC-PINN module leads to substantial improvements in the model’s accuracy, robustness, and convergence rate. By balancing the computational complexity and performance, BC-PINN provides an efficient and reliable solution for lesion segmentation. Figure 3C displays the architectural composition of the BC-PINN module.

Physics-informed Neural Networks [31] directly embed the physical domain knowledge (such as differential equations, boundary conditions, etc.) into the network training process so that the model can follow the physical laws while learning the data distribution. On this basis, we propose the BC-PINN module, which not only retains the core idea of PINN embedding physical prior knowledge but also optimizes the design for image segmentation tasks. By introducing spatial boundary constraints in the decoding phase, BC-PINN ensures that the predicted results comply with the spatial boundary conditions of the physical scene, thereby enhancing the model’s generalization ability and prediction accuracy.

First, the input prediction results are expressed as four-dimensional tensors:

(11) $$P\in {\mathbb{R}}^{B\times C\times H\times W}$$

where B is the batch size, C is the number of channels (usually 1), and H and W respectively represent the height and width of the prediction chart. For the pixel (b,c,y,x) whose prediction probability is greater than the threshold (such as 0.5), we extract its spatial coordinates for subsequent boundary constraint calculation. The extraction coordinate formula is as follows:

(12) $$C=\left\{(b,c,y,x)|P_{b,c,y,x}>0.5\right\}$$

Then, based on the image size (H,W), we construct four kinds of spatial penalty terms, which correspond to the four directions where the prediction point is out of bounds: left, up, right, and down. Specifically, for each prediction point the equation is as follows:

(13) $$\left\{\begin{array}{l} x_{\min}=ReLU(-x)\\ y_{\min}=ReLU(-y)\\ x_{\max}=ReLU(x-(W-1))\\ y_{\max}=ReLU(y-(H-1)) \end{array}\right.$$

where ReLU(⋅) is a modified linear unit function to ensure that the penalty value is non-negative. The above four items, respectively, represent the distance of the prediction point beyond the left, upper, right, and lower boundaries of the image.

Then, the penalty terms of all exceeding points are accumulated and normalized to the PINN loss:

(14) $$L_{\mathrm{PINN}}=\frac{1}{B\cdot H\cdot W}\sum_{(b.c.y.x)\in C}\left(x_{\min}+y_{\min}+x_{\max}+y_{\max}\right)$$

This penalty term constrains the prediction mask in space and explicitly suppresses the prediction beyond the image boundary. In the training process, it promotes the model to generate prediction results in line with the spatial range of the image through automatic derivation and back propagation.

At the same time, in order to take into account the segmentation accuracy and spatial constraints, the BC-PINN module comprehensively considers Dice loss, BCE loss, and PINN loss in the total loss to form the final polynomial loss function:

(15) $$L_{total}=w_{dice}\cdot L_{dice}+w_{BCE}\cdot L_{BCE}+w_{PINN}\cdot L_{PINN}.$$

Among them, $w_{dice}$, $w_{BCE}$ and $w_{PINN}$ are the weight coefficients of the three parts of loss, respectively. Dice loss and BCE loss jointly measure the similarity between the prediction and the real label, while PINN loss improves the segmentation quality through space restriction.

In the BC-PINN module, the boundary physical constraints are not only reflected in the loss calculation stage but also the prediction decoding process. Unlike the conventional segmentation task, which only relies on the direct comparison between the prediction probability and the label, BC-PINN introduces spatial location information according to the prediction distribution in the decoding process to ensure that the decoding results not only reflect the distribution of data characteristics but also follow the physical laws of spatial boundaries. In other words, the BC-PINN module guides the model to learn reasonable physical boundary information in training by evaluating the boundary position of the prediction mask. Even in the case of limited labeled samples, the model can also output more reasonable prediction results with the help of physical constraints.

The introduction of the BC-PINN module not only enhances the boundary perception ability of the model but also makes full use of the knowledge of the physical field, so that the network prediction not only depends on the data characteristics but also follows the physical laws. In the case of limited annotation data or uncertainty, BC-PINN can effectively improve the prediction stability and generalization performance through physical constraints and avoid model over-fitting. In addition, through the boundary penalty processing of the prediction mask, the BC-PINN module significantly reduces the abnormal prediction and improves the reliability and interpretability of the model in practical applications. For the problem of irregular growth and complex texture of disease spots, BC-PINN can better capture the subtle changes and complex boundaries of disease spots. This method overcomes the limitations of traditional data-driven models in dealing with irregular lesion morphology and provides more reliable and accurate prediction results. The “Effectiveness of the BC-PINN” section is analyzed experimentally considering the validity of BC-PINN.

## 3. Results and Analysis

This section verifies the performance of KBNet through a series of experiments, focusing on its advantages in solving multi-scale disease spots and irregular growth of disease spots. The specific experimental contents include (a) introducing the hardware and software environment of the experiment; (b) evaluating the experimental performance index of KBNet; (c) analyzing the contribution and effectiveness of each module of KBNet in the overall performance; (d) ablation experiments used to assess the impact of each module on the model’s performance; (e) comparison with other advanced segmentation methods (KBNet shows its competitiveness in the task of rice disease segmentation); (f) visually evaluating the segmentation ability of KBNet through visualization results; (g) conducting a generalization experiment to evaluate the adaptability and robustness of KBNet on different datasets; and (h) evaluation of KBNet’s performance across different rice disease categories to assess its effectiveness and robustness.

### 3.1. Experimental Environment

In order to ensure the accuracy and repeatability of the experimental results, all experiments were carried out in a unified hardware and software environment. The main hardware devices used in this experiment include NVIDIA GeForce RTX 4090 and 16 vCPU Intel (R) Xeon (R) Platinum 8352v CPU @ 2.10 GHz CPU. Although the specific versions of Python, CUDA, and CUDNN have no impact on the experimental results, ensuring the compatibility of the software and hardware is very important for the smooth progress of the experiment. The hardware configuration used in the experiment is provided by the AutoDL platform, which ensures the consistency and stability of the experiment. On this basis, we implemented KBNet based on CUDA 11.1 and PyTorch 1.8.1. The hardware details and software settings are provided in Table 1.

### 3.2. Evaluation Indicators

To assess the model’s performance comprehensively, four evaluation metrics—IoU, Dice [32], Accuracy, and Recall—were applied, as described in Equations (16)–(19). Four region categories were used for precision: TP represents true positives, TN stands for true negatives, FP indicates false positives, and FN denotes false negatives.

IoU quantifies the disparity between the ground truth and predicted regions, reflecting the overlap ratio between the rice disease areas and their corresponding labels:

(16) $$IoU=\frac{TP}{TP+FN+FP}\times 100\%$$

The Dice coefficient is a metric used to measure similarity and is commonly applied to assess the resemblance between two samples:

(17) $$Dice=\frac{2TP}{\left(TP+FN)+(TP+FP\right)}\times 100\%.$$

Accuracy reflects the proportion of rice leaf images that are segmented correctly compared to the total number of samples, encompassing both correct and erroneous segmentations:

(18) $$Accuracy=\frac{TP+TN}{TP+FP+TN+FN}\times 100\%$$

Recall gauges the number of positive samples that were accurately predicted:

(19) $$Recall\;=\frac{TP}{TP+FN}\times 100\%$$

### 3.3. Module Effectiveness Experiments

#### 3.3.1. Effectiveness of the KF-KAN

In order to effectively solve the difficulties in multi-scale lesion processing, this paper proposes a new feature extraction module—KF-KAN. This module combines KANs and Kalman filters to improve the efficiency of multi-scale lesion feature extraction. KANs can effectively model complex nonlinear relationships and enhance the expression of lesion features, while the Kalman filter improves the accuracy and stability of multi-scale lesion features through a dynamic feature update mechanism. In addition, KF-KAN further enhances the robustness of the model by introducing the adaptive residual structure and Dropout layer, ensuring efficient performance in complex environments. In order to verify the effectiveness of the KF-KAN module, we compared it with several feature extraction modules such as AIFI [33], MFF [34], BiFPN [35], VMamba [36], MDFM [37] and Densenet [38]. See Table 2 for the test results. The results showed that KF-KAN performed well in all evaluation indexes, especially in the treatment of multi-scale lesions. In contrast, although AIFI can deal with multi-scale disease spots within a certain range, it is difficult to capture the features of small-scale disease spots because of its low accuracy in dealing with the details of disease spots. The MFF module performs well in multi-scale feature fusion, but its effect in noise suppression is weak. BiFPN enhances the diversity of features through multi-path information transmission, but it is still difficult to maintain efficient feature extraction in a large range, especially in the extraction of complex lesions. VMamba and MDFM have strong ability in the feature extraction of disease spots, but when faced with high noise data, the stability decreases, leading to performance fluctuations. Although Densenet can enhance feature propagation through dense connections, it has limited ability in fine-grained modeling of multi-scale lesions. To sum up, the KF-KAN module is more suitable for feature extraction of multi-scale disease spots. Combined with the optimal design of KANs and the Kalman filter, it performs well in multi-scale disease spot extraction and noise suppression. At the same time, the introduction of adaptive residual structure and the Dropout layer makes the model segmentation more accurate.

#### 3.3.2. Effectiveness of the BC-PINN

In order to solve the problem of irregular growth and complex texture in the segmentation model, this paper proposes the BC-PINN module. By introducing the penalty term of the prediction mask and image boundary in the training process, the module constrains the spatial position of prediction results and ensures that the model can more accurately locate the lesion area. In addition, by adaptively adjusting the weight of physical information, BC-PINN effectively ensures that the prediction results conform to the boundary conditions of the physical scene, thus improving the generalization ability and prediction rationality of the model. By embedding physical information such as the growth law of the lesion into the loss function, BC-PINN can guide the neural network to segment the lesion more accurately. To evaluate the effectiveness of the BC-PINN module, we compared it with several existing loss functions, including Focal [39], Inner-IoU [40], WIoUv3 [41], Tversky [42] and Hinge [43]. As shown in Table 2, the experimental results are provided. BC-PINN shows superior effects on several key performance indicators. Specifically, BC-PINN significantly improves the segmentation accuracy of the model by introducing a boundary penalty in the training process. Compared with other methods, BC-PINN not only performs better in accuracy but also improves the stability and convergence speed of model training. Through optimization design, the BC-PINN module has become an indispensable core component in the KBNet framework, providing more efficient and accurate technical support for disease segmentation.

### 3.4. Ablation Experiment

Four ablation experiments were carried out on the self-constructed lesion dataset in this section to validate the impact of adding the KF-KAN and BC-PINN modules on model performance. The experiments were carried out under the conditions of control variables. The results were recorded in Table 3 and were analyzed and compared in detail. In this study, the KF-KAN module aims to improve the extraction accuracy of multi-scale lesion features and combines KANs and Kalman filters. By optimizing the feature extraction strategy, the interference of redundant information is effectively reduced, and the accurate extraction ability of the lesion region is significantly improved. The experimental results show that after adding the KF-KAN module, the Dice coefficient increases by 3.1%, and IoU increases by 5.1%. This module shows significant advantages in dealing with complex backgrounds and multi-scale lesion features. By introducing physical information constraints, the BC-PINN module optimizes the modeling of the disease spot growth law and helps the model maintain high segmentation accuracy when dealing with irregular disease spot boundaries and complex textures. The experimental results indicate that, when compared to the baseline model, BC-PINN enhances the IoU by 5.4% and the Dice coefficient by 4%. This module is especially suitable for the processing of complex lesion morphology and effectively improves the segmentation accuracy and model robustness. The combination of the KF-KAN and BC-PINN modules leads to a further improvement in the model’s performance. Results from the experiments reveal that the combined model’s Dice coefficient and IoU are 83.9% and 72.3%, respectively, which are 6.1% and 8.3% greater than those of the baseline model. This result proves the complementarity of KF-KAN and BC-PINN modules and the effectiveness of their combination in improving segmentation accuracy and enhancing model stability. Through these four ablation experiments, we verified the important role of KF-KAN and BC-PINN modules in the task of lesion segmentation. The integration of the two modules not only boosts the model’s segmentation accuracy but also strengthens its robustness in complex backgrounds, further demonstrating the effectiveness and benefits of these modules in disease spot segmentation.

### 3.5. Experiment Comparing KBNet with Other Models

In order to further analyze the performance of the KBNet model, we compared several traditional and current advanced single-mode and multi-mode segmentation methods on the same dataset. Table 4 shows the test results of each model. First, we compare several popular single-mode segmentation methods, including UNet, UNet++, DeepLabv3+, and SegNet. With its simple and efficient encoder–decoder structure, UNet has gained widespread application in medical image segmentation and various other tasks. However, when dealing with complex backgrounds or tasks with multi-scale features, UNet is prone to losing high-level context information, which affects the segmentation accuracy. UNet++ optimizes feature fusion by introducing jump connections, which significantly improve the segmentation ability of multi-scale images. However, due to its more complex network structure, the training and debugging process is cumbersome. DeepLabv3+ can better capture the global information by introducing a hole convolution to expand the receptive field. However, due to its deeper network structure, the convergence speed in the training process is slow, and the processing effect for some simple scenes may not be as good as expected. SegNet extracts features using an encoder–decoder architecture with pixel-wise softmax, which enhances the model’s ability to capture fine-grained details in segmentation tasks. However, due to its reliance on deep feature extraction and decoding layers, the model can be computationally intensive and may struggle with more complex segmentation scenarios. Compared with the traditional single-mode segmentation network, the segmentation accuracy of the multi-modal model has been significantly improved, especially when dealing with multiple scattered tiny points. In the multi-modal segmentation task, we compared CLIP, GLoRIA, and LViT. CLIP can improve the accuracy of the model in some tasks by combining visual and textual information, especially in scenes that need to understand complex semantics. However, CLIP is highly dependent on text data, and its generalization ability between different datasets or tasks may be limited. GLoRIA enhances the adaptability of the model to complex scenes by optimizing the modeling of visual and semantic relationships, but its heavy dependence on semantic data limits the scope of application of the model, and in some scenes, the effect of feature fusion is not as good as expected. LViT combines the advantages of a visual converter and a convolutional neural network and performs well in image segmentation, especially when dealing with details and complex textures, but its processing of low-quality images is relatively weak, and it is vulnerable to high-frequency light interference when processing rice disease images. The experimental results show that KBNet is 12.1%, 6.7%, 1.5%, and 9.6% higher than UNet, UNet++, DeepLabv3+, and SegNet on IoU, and 10.5%, 5.4%, 1.3%, and 5.1% higher than Dice. Compared with other multi-modal segmentation methods, KBNet achieves better performance than CLIP, GLoRIA, and LViT, with 3.5%, 6.8%, and 8.3% improvement on IoU, and 2.5%, 7.3%, and 6.1% improvement on Dice, respectively. To sum up, KBNet is superior to most existing segmentation networks in overall performance, especially in the application of multi-modal feature fusion, showing excellent performance. We believe that the advantages of the KBNet model stem from the following points: (a) KBNet combines visual and semantic information to make up for the limitations of a single visual information. (b) Through the KF-KAN module, combined with KANs and the Kalman filter, the feature extraction strategy is optimized to significantly improve the accurate extraction ability of the lesion area. (c) By introducing physical information constraints, the BC-PINN module optimizes the modeling of the disease spot growth law, helping the model maintain high segmentation accuracy when dealing with irregular disease spot boundaries and complex textures. (d) The custom dataset created in this research removes many blurred and low-resolution images, which facilitates better model training.

### 3.6. Comparison of the Visualization Outcomes

Table 5 shows how we can visualize the segmentation results of LViT and KBNet to better understand KBNet. We use different methods to segment three diseases, namely Bacterial Blight, Blast, and Tungro.

In group A, we performed segmentation experiments on typical lesion areas of Bacterial Bright. The experimental results show that the LViT model still has a certain degree of fuzziness in the location of the lesion edge, especially in the area with thin and dense lesions, which is prone to edge fracture. In contrast, KBNet is obviously superior to the contrast method in detail recognition and can completely and accurately reconstruct the shape of the lesion. This performance improvement is mainly due to the introduction of the KF-KAN module, which combines the advantages of KANs in nonlinear modeling and the dynamic state update ability of the Kalman filter and significantly enhances the perception ability of the model to complex textures and small lesions.

In group B, we selected the Blast image sample with a blurred boundary. The experiment found that LViT was prone to the problems of false segmentation or regional adhesion when facing the lesions with edge diffusion and unfixed shape. KBNet, by introducing the BC-PINN module, embeds the prior knowledge of the lesion growth mechanism into the loss function so that the model has the ability to accurately model the non-rigid lesion structure. At the same time, the addition of a boundary penalty mechanism further restricts the segmentation edge, improves the resolution of the model to the lesion boundary, and makes KBNet perform better than other methods in the fuzzy edge area.

In group C, we used images containing Tungro lesions and complex backgrounds for testing. The results show that KBNet still shows stable and excellent segmentation performance and can accurately capture the main area of the disease spot in the complex interference background while suppressing background misjudgment. The efficient fusion of local and global features by KF-KAN and the modeling of spatial diffusion characteristics by BC-PINN enable KBNet to still maintain strong adaptability and robustness in the real field environment.

To sum up, through the synergy of KF-KAN and BC-PINN, KBNet not only achieves stronger feature expression ability and boundary perception ability but also significantly optimizes the overall quality of disease segmentation, providing reliable support for subsequent intelligent disease diagnosis.

### 3.7. Generalization Experiments

In order to further evaluate the effectiveness of KBNet, we tested the performance of the model on different crops and disease types. First, we selected the Plantvillage maize dataset, which mainly contains 1044 finely labeled images of Northern Leaf Blight and 1044 corresponding text-marked sentences generated by a special marking software. To ensure the precision of the experiment, we divided the dataset into 732 training set entries, 204 test set entries, and 108 verification set entries. The maize dataset covers the typical symptoms of northern leaf blight, and its performance under different environmental conditions is highly representative. Therefore, the dataset can simulate the maize disease in the real scene to a certain extent. In addition, we also selected the public tomato dataset of Plantvillage, which covers four common types of tomato diseases: Septoria Leaf Spot, Early Blight, Leaf Mold, and Late Blight, with a total of 1300 images. We split the image into a training set, test set, and validation set with a ratio of 9:2:2, which enables us to verify the generalization ability and segmentation accuracy of the model on a wider range of disease types. During the experiment, we used the above dataset to verify the performance of KBNet in the segmentation task of different crop diseases; the outcomes are presented in Table 6. For the maize dataset, the model can accurately segment the northern leaf blight, with a Dice coefficient of 87.7% and IoU of 78.5%. For the tomato dataset, the model is stable in dealing with a variety of diseases, with a Dice coefficient of 85.0% and an IoU of 74.1%. These results show that our model can maintain high accuracy in the face of a variety of different crops and disease types, indicating its high versatility.

### 3.8. Performance Evaluation for Each Disease Category

To evaluate the performance of KBNet across different rice disease types, we conducted segmentation experiments using three distinct rice disease datasets: Blast, Bacterial Blight, and Tungro. These datasets consist of images with specific disease symptoms, allowing us to assess the model’s ability to handle a variety of diseases within the same crop. The Blast dataset includes 522 images, with 302 for training, 150 for validation, and 70 for testing. The Bacterial Blight dataset has 518 images, divided into 297 for training, 150 for validation, and 71 for testing. Lastly, the Tungro dataset contains 510 images, with 289 for training, 150 for validation, and 71 for testing. The performance of KBNet on these datasets is presented in Table 7. In the experiments, KBNet shows excellent segmentation results for Blast, achieving a Dice coefficient of 90.9%, an IoU of 83.3%, a Recall of 80.2%, and an Accuracy of 79.5%. The model performs reasonably well on Tungro, with a Dice of 86.7%, an IoU of 76.7%, a Recall of 78.9%, and an Accuracy of 68.0%. However, the performance on Bacterial Blight is comparatively lower, with a Dice of 74.1%, an IoU of 56.9%, a Recall of 69.8%, and an Accuracy of 49.3%. These results indicate that KBNet performs the best on Blast due to clearer disease characteristics, while the Bacterial Blight presents more challenges, with the main reason for its decreased performance being that the lesions of Bacterial Blight often mix with leaf veins or naturally aging areas. Moreover, the images of Bacterial Blight exhibit higher intra-class variability and more complex backgrounds. Overall, these experiments demonstrate that KBNet performs robustly across different rice disease categories, showcasing its effectiveness in disease segmentation tasks. The model’s performance varies slightly depending on the type of disease, but it maintains a strong ability to generalize across different rice disease categories.

## 4. Discussion

In this study, we propose a new rice disease segmentation method, which is significantly improved compared with the existing methods. The proposed KBNet model combines multiple innovative modules to make it perform well in complex agricultural scenarios. Specifically, on the basis of the LViT model, we propose the KF-KAN module. This module can efficiently capture the nonlinear features of different scales through KAN and dynamically update and fuse the multi-scale information combined with a Kalman filter so as to achieve accurate feature extraction of complex lesion areas. In addition, we introduce the BC-PINN module, which combines the prior knowledge of physical laws with the powerful modeling ability of a neural network. At the same time, the accuracy of edge segmentation is further improved through the boundary penalty mechanism. We believe that the advantages of the KBNet model come from the following points: (a) KBNet combines visual and semantic modes, making full use of the complementarity and correlation between modes. (b) The KF-KAN module can significantly improve the accurate extraction ability of multi-scale disease spots. (c) The BC-PINN module helps the model maintain high segmentation accuracy when dealing with irregular lesion boundaries and complex textures.

Compared with existing methods, such as AISOA-SSformer proposed by Dai et al., this method also uses the architecture based on transformer for segmentation. AISOA-SSformer proposes a sparse global update perceptron and combines several optimization techniques, such as the annealing-integrated sparrow optimization algorithm, to enhance the segmentation performance of the model in complex environments. Although AISOA-SSformer has made significant progress in improving segmentation accuracy, KBNet can provide higher accuracy segmentation results when processing irregular lesion boundaries by combining visual information with physical knowledge through the BC-PINN module, which is difficult for other models. Compared with the LVF proposed by Hu et al., KBNet also shows its unique advantages. LVF enhances the processing ability of image features by introducing the Randomized Impact Fusion Network and the GCM-enhanced feature network, especially in high-light and complex background environments. However, when the lesions show multi-scale changes, the performance of LVF is limited. In contrast, KBNet can effectively deal with irregular lesions through the KF-KAN module, especially when dealing with small lesions and blurred boundaries, showing a better segmentation effect. In a word, KBNet can achieve high accuracy in rice disease segmentation, surpass the traditional methods, and provide a promising tool for real-time agricultural disease management.

**Figure 4:**
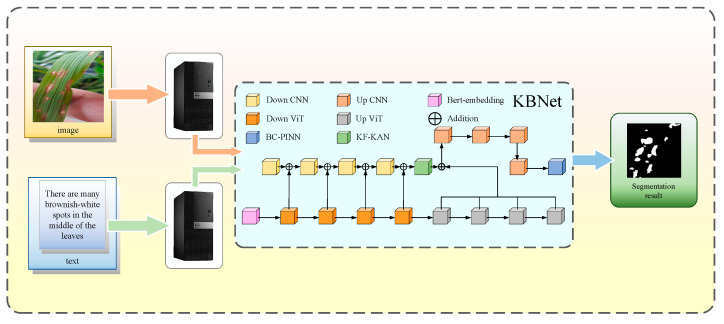
Workflow for a practical application.

To evaluate the performance of KBNet, we established a rice disease segmentation system, which aims to accurately segment the rice disease through the image and the corresponding text description. In the research stage, we collected the images of rice leaves containing disease. First, upload the rice disease leaf image, and the system will automatically adjust the image size to 224 × 224 to meet the input requirements of the model. Then, enter the description information corresponding to the disease image in the text box, and the system will carry out feature extraction and transformation according to these descriptions. After the dual input of image and text information into the KBNet model, the final segmentation results are presented through the visualization platform to help users evaluate the type and scope of disease in detail. Figure 4 shows the whole workflow.

**Figure 5:**
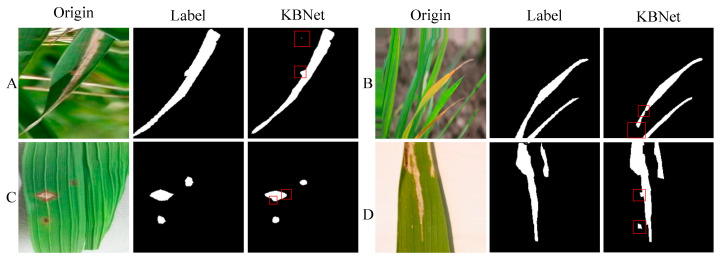
The segmentation results of practical applications. Among them, (**A**–**D**) are the segmentation results of four different images.

Following the implementation of the system, we tested and deployed it in real-world scenarios to assess its performance. In practical applications, the KBNet model has demonstrated notable advantages in rice disease segmentation tasks. However, as shown in Figure 5, the model also faces certain limitations. While the system performs well in segmenting most disease areas, some issues arise in specific situations. For example, the model may miss detection or produce incomplete segmentation in small or unclear disease areas. Secondly, in some low-contrast or irregular disease areas, the segmentation accuracy may be affected, resulting in unclear boundaries, which will affect the accuracy of the overall recognition. Moreover, for some special lesions, the model may have a slight deviation in segmentation. Especially when the edge of the lesion is damaged, the model may slightly underestimate or over-segment specific areas. Finally, although the model has been able to better deal with the complex background and details in the image, when facing the image with high complexity or different disease types, the model may also be disturbed by the background, resulting in the false segmentation of some small areas of disease spots, affecting the overall effect. Looking forward to the future, our research work will give priority to the following objectives: (1) The existing datasets have limited samples in the complex environment and early disease stage, which makes it difficult to meet the generalization requirements of the model in the actual scene. In the future, we will focus on collecting data under different lighting, background, and growth period conditions to improve the segmentation ability of the model for diversified diseases. (2) In order to improve the application ability of the model in the actual agricultural scene, we will promote the lightweight optimization of the model, reduce the demand for computing resources through structural compression and parameter simplification, and realize the real-time detection and rapid response of rice diseases. (3) In terms of text modality, we will strengthen the text coding model and combine the agricultural domain knowledge map and structured semantic modeling to improve the model’s understanding of disease description and the accuracy of cross-modality fusion. Through the above optimization, our goal is to develop a more precise, robust, and universally adaptable multimodal rice disease segmentation system, which will offer substantial support for smart monitoring and targeted prevention and control of agricultural diseases.

## 5. Conclusions

In order to explore the best method of rice leaf disease segmentation, we propose an image- and text-based rice disease segmentation method using KBNet. Firstly, we design the KF-KAN module, which can accurately extract the features of complex disease spots, especially in multi-scale disease areas. Then, we propose the BC-PINN module. BC-PINN can effectively constrain the prediction of irregular growth lesions and use physical information to generate reliable monitoring signals so as to further optimize the segmentation effect. Through these innovative module designs, our model has achieved significant improvement in the task of rice disease segmentation, showing high accuracy and robustness. This study can help producers identify rice leaf diseases in a timely and accurate manner and take targeted control measures. It provides a new reference for the application of deep learning in modern rice agriculture. During the experiment, we divided the 1550 images into a 4:2:1 split, comprising the training set, verification set, and test set, respectively. Under the same experimental conditions, compared with LViT, the IoU and dice coefficients of KBNet are increased by 8.3% and 6.1%, respectively. Compared with the current mainstream split network pairs, KBNet’s IoU is 72.3%, Dice is 83.9%, Recall is 76.3%, and Accuracy is 65.6%. This approach demonstrates outstanding performance, not just in segmentation accuracy, but also in handling multi-scale disease spots and the irregular growth of these spots.

This research provides important technical support for intelligent agriculture and automation in disease control. It lays a theoretical foundation for the promotion of precision agriculture technology. By improving disease identification and segmentation, it aims to enhance rice disease prevention and control, contributing to global food security and sustainable agricultural development. In the future, this technology will promote intelligent agricultural practices, reduce pesticide usage, and mitigate environmental impact. We also plan to extend this technology to other crops and continue advancing AI-based solutions for agricultural challenges. Despite the challenges, we believe that through continuous technological breakthroughs and optimization, this technology will play an increasingly important role in promoting agricultural intelligence and improving agricultural productivity.

## Figures and Tables

**Table 1:** Configuration of software and hardware environments.

Hardware environment	GPU	NVIDIA GeForce RTX 4090
	CPU	16 vCPU Intel(R) Xeon(R) Platinum 8352 V CPU @ 2.10 GHz
	RAM	120 GB
	Video memory	24 GB
Software environment	CUDNN	V8.0.5
	CUDA Toolkit	V11.1
	OS	Ubuntu 18.04
	Pytorch	1.8.1
	Python	3.8

**Table 2:** Results of the KBNet module experiment.

Model	Method	IoU	Dice	Recall	Accuracy
KF-KAN	AIFI	58.4%	71.1%	61.7%	55.4%
	MFF	61.7%	76.3%	68.1%	57.9%
	BiFPN	60.1%	74.4%	67.6%	55.5%
	VMamba	63.2%	75.6%	65.4%	58.4%
	MDFM	66.8%	79.5%	71.9%	64.1%
	Densenet	59.4%	73.7%	62.7%	53.0%
	Ours	69.1%	80.9%	74.2%	65.4%
BC-PINN	Focal	54.2%	67.7%	63.3%	52.2%
	Inner-IoU	61.3%	74.3%	68.3%	56.1%
	WIoUv3	68.2%	81.1%	69.8%	60.5%
	Tversky	53.7%	66.3%	59.9%	48.2%
	Hinge	66.7%	78.6%	66.6%	57.3%
	Ours	69.4%	81.8%	70.7%	63.1%

**Table 3:** Ablation experiments.

KF-KAN	BC-PINN	IoU	Dice	Recall	Accuracy
--	--	64.0%	77.8%	73.2%	61.3%
√		69.1%	80.9%	74.2%	65.4%
	√	69.4%	81.8%	70.7%	63.1%
√	√	72.3%	83.9%	76.3%	65.6%

**Table 4:** Comparative experiment results for different modes.

Method	IoU	Dice	Recall	Accuracy	FPS ^a^	IoU Standard Deviation ^b^
UNet	60.2%	73.4%	73.1%	55.1%	11.12	5.31%
UNet++	65.6%	78.5%	78.3%	62.5%	8.79	3.60%
DeepLabv3+ [44]	70.8%	82.6%	81.7%	84.6%	7.42	5.04%
SegNet [45]	62.7%	78.8%	72.5%	66.1%	13.30	9.21%
CLIP [46]	68.8%	81.4%	74.6%	61.7%	17.52	2.37%
GLoRIA [47]	65.5%	76.6%	69.8%	63.8%	8.22	6.65%
LViT	64.0%	77.8%	73.2%	61.3%	9.34	1.97%
Ours	72.3%	83.9%	76.3%	65.6%	9.19	1.44%

^a^ FPS stands for “Frames Per Second,” a metric used to measure the speed at which a computer or processor can process image frames per second. It reflects the efficiency of the system in handling images, videos, or training data. ^b^ IoU Standard Deviation quantifies the variability of a model’s segmentation performance across validation samples, calculated as the dispersion of Intersection over Union (IoU) values around their mean. A lower value indicates more consistent predictions, while a higher value suggests sensitivity to data variations or instability in the model or validation set.

**Table 5:** Comparison of visualization test results.

Method		Segmentation Results	
Image	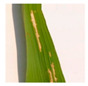	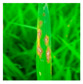	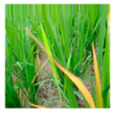
Ground truth	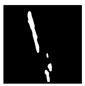	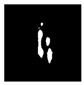	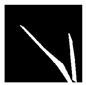
LViT	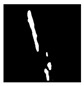	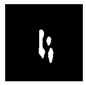	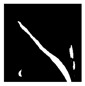
KF-KAN	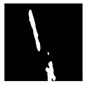	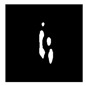	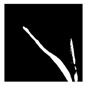
BC-PINN	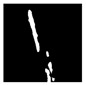	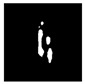	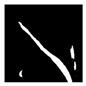
KBNet	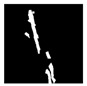	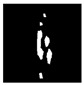	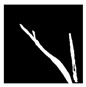
	A	B	C

**Table 6:** KBNet’s segmentation results across different public datasets.

Dataset	IoU	Dice	Recall	Accuracy
PlantVillage Maize	78.5%	87.7%	79.1%	74.9%
PlantVillage Tomato	74.1%	85.0%	75.9%	69.2%

**Table 7:** The segmentation results of different types of rice diseases.

Dataset	IoU	Dice	Recall	Accuracy
Blast	83.3%	90.9%	80.2%	79.5%
Bacterial Blight	56.9%	74.1%	69.8%	49.3%
Tungro	76.7%	86.7%	78.9%	68.0%

## Data Availability

The datasets used in this paper are available on demand.

## References

[1] Bozarovich K.J., Farxod ogli B.S., Abdivaitovich A.S. (2023). Rice Growth and Development. Zenodo (CERN Eur. Organ. Nucl. Res.).

[2] Tang Z., He X., Zhou G., Chen A., Wang Y., Li L., Hu Y. (2023). A Precise Image-Based Tomato Leaf Diseases Detection Approach Using PLPNet. Plant Phenomics.

[3] Savary S., Willocquet L., Pethybridge S.J., Esker P., McRoberts N., Nelson A. (2019). The Global Burden of Pathogens and Pests on Major Food Crops. Nat. Ecol. Evol..

[4] Li M., Zhou G., Chen A., Yi J., Lu C., He M., Hu Y. (2022). FWDGAN-Based Data Augmentation for Tomato Leaf Disease Identification. Comput. Electron. Agric..

[5] Dai W., Zhu W., Zhou G., Liu G., Xu J., Zhou H., Hu Y., Liu Z., Li J., Li L. (2024). AISOA-SSformer: An Effective Image Segmentation Method for Rice Leaf Disease Based on the Transformer Architecture. Plant Phenomics.

[6] Deng J., Huang W., Zhou G., Hu Y., Li L., Wang Y. (2023). Identification of Banana Leaf Disease Based on KVA and GR-ARNet1. J. Integr. Agric..

[7] Ronneberger O., Fischer P., Brox T. (2015). U-net: Convolutional networks for biomedical image segmentation. Proceedings of the Medical Image Computing and Computer-Assisted Intervention–MICCAI 2015: 18th International Conference.

[8] Chen L.-C., Papandreou G., Kokkinos I., Murphy K., Yuille A.L. (2018). DeepLab: Semantic Image Segmentation with Deep Convolutional Nets, Atrous Convolution, and Fully Connected CRFs. IEEE Trans. Pattern Anal. Mach. Intell..

[9] He K., Gkioxari G., Dollar P., Girshick R. Mask R-CNN. Proceedings of the 2017 IEEE International Conference on Computer Vision (ICCV).

[10] Deng Y., Xi H., Zhou G., Chen A., Wang Y., Li L., Hu Y. (2023). An Effective Image-Based Tomato Leaf Disease Segmentation Method Using MC-UNet. Plant Phenomics.

[11] Chua L.O. (1997). CNN: A Vision of Complexity. Int. J. Bifurc. Chaos.

[12] Liu Z., Zhou G., Zhu W., Chai Y., Li L., Wang Y., Hu Y., Dai W., Liu R., Sun L. (2024). Identification of Rice Disease under Complex Background Based on PSOC-DRCNet. Expert Syst. Appl..

[13] Zhang S., Zhang C. (2023). Modified U-Net for Plant Diseased Leaf Image Segmentation. Comput. Electron. Agric..

[14] Lu Y., Qin X., Fan H., Lai T., Li Z. (2021). WBC-Net: A White Blood Cell Segmentation Network Based on UNet++ and ResNet. Appl. Soft Comput..

[15] Zhou Z., Siddiquee M.M.R., Tajbakhsh N., Liang J. (2018). UNet++: A Nested U-Net Architecture for Medical Image Segmentation. Deep Learning in Medical Image Analysis and Multimodal Learning for Clinical Decision Support.

[16] Cai C., Wang Q., Cai W., Yang Y., Hu Y., Li L., Wang Y., Zhou G. (2023). Identification of Grape Leaf Diseases Based on VN-BWT and Siamese DWOAM-DRNet. Eng. Appl. Artif. Intell..

[17] Li M., Zhou G., Chen A., Li L., Hu Y. (2023). Identification of Tomato Leaf Diseases Based on LMBRNet. Eng. Appl. Artif. Intell..

[18] Hu Y., Zhu J., Zhou G., He M., Lv M., Wang J., Chen A., Deng J., Jiang Y. (2024). LVF: A Language and Vision Fusion Framework for Tomato Diseases Segmentation. Comput. Electron. Agric..

[19] Li J., Zhao F., Zhao H., Zhou G., Xu J., Gao M., Li X., Dai W., Zhou H., Hu Y. (2024). A Multi-Modal Open Object Detection Model for Tomato Leaf Diseases with Strong Generalization Performance Using PDC-VLD. Plant Phenomics.

[20] Tuncer T., Dogan S., Subasi A. (2021). EEG-Based Driving Fatigue Detection Using Multilevel Feature Extraction and Iterative Hybrid Feature Selection. Biomed. Signal Process. Control.

[21] Chavan V., Shah J., Vora M., Vora M., Verma S. (2020). A Review on Feature Extraction Methods for Image Analysis. SSRN Electron. J..

[22] Bradley A.P. (2003). Shift-Invariance in the Discrete Wavelet Transform. Digit. Image Comput. Tech. Appl..

[23] Xu M., Ma Q., Zhang H., Kong D., Zeng T. (2024). MEF-UNet: An End-To-End Ultrasound Image Segmentation Algorithm Based on Multi-Scale Feature Extraction and Fusion. Comput. Med. Imaging Graph..

[24] Borse S., Wang Y., Zhang Y. (2021). Fatih Porikli InverseForm: A Loss Function for Structured Boundary-Aware Segmentation. arXiv.

[25] Bougourzi F., Dornaika F., Nakib A., Taleb-Ahmed A. (2024). Emb-Trattunet: A Novel Edge Loss Function and Transformer-CNN Architecture for Multi-Classes Pneumonia Infection Segmentation in Low Annotation Regimes. Artif. Intell. Rev..

[26] Li A., Jiao L., Zhu H., Li L., Liu F. (2022). Multitask Semantic Boundary Awareness Network for Remote Sensing Image Segmentation. IEEE Trans. Geosci. Remote Sens..

[27] (2019). Kaggle, PlantVillage Dataset. https://www.kaggle.com/datasets/abdallahalidev/plantvillage-dataset.

[28] Li Z., Li Y., Li Q., Wang P., Guo D., Lu L., Jin D., Zhang Y., Hong Q. (2024). LViT: Language Meets Vision Transformer in Medical Image Segmentation. IEEE Trans. Med. Imaging.

[29] Liu Z., Wang Y., Vaidya S., Ruehle F., Halverson J., Soljacic M., Hou T.Y., Tegmark M. (2024). KAN: Kolmogorov-Arnold Networks. arXiv.

[30] Welch G., Bishop G. (1995). An Introduction to the Kalman Filter.

[31] Hu H., Qi L., Chao X. (2024). Physics-Informed Neural Networks (PINN) for Computational Solid Mechanics: Numerical Frameworks and Applications. Thin-Walled Struct..

[32] Guindon B., Zhang Y. (2016). Application of the Dice Coefficient to Accuracy Assessment of Object-Based Image Classification. Can. J. Remote Sens..

[33] Zhao Y., Lv W., Xu S., Wei J., Wang G., Dang Q., Liu Y., Chen J. DETRs Beat YOLOs on Real-Time Object Detection. Proceedings of the 2022 IEEE/CVF Conference on Computer Vision and Pattern Recognition (CVPR).

[34] Fang S., Wang Y., Zhou G., Chen A., Cai W., Wang Q., Hu Y., Li L. (2022). Multi-Channel Feature Fusion Networks with Hard Coordinate Attention Mechanism for Maize Disease Identification under Complex Backgrounds. Comput. Electron. Agric..

[35] Wang X., Zhang X., Zhou N. Improved YOLOv5 with BiFPN on PCB Defect Detection. Proceedings of the 2021 2nd International Conference on Artificial Intelligence and Computer Engineering (ICAICE).

[36] Xu R., Yang S., Wang Y., Du B., Chen H. (2024). A Survey on Vision Mamba: Models, Applications and Challenges. arXiv.

[37] Luo F., Zhou T., Liu J., Guo T., Gong X., Ren J. (2023). Multiscale Diff-Changed Feature Fusion Network for Hyperspectral Image Change Detection. IEEE Trans. Geosci. Remote Sens..

[38] Roy A.M. (2023). Jayabrata Bhaduri DenseSPH-YOLOv5: An Automated Damage Detection Model Based on DenseNet and Swin-Transformer Prediction Head-Enabled YOLOv5 with Attention Mechanism. Adv. Eng. Inform..

[39] Lin T.-Y., Goyal P., Girshick R., He K., Dollar P. (2018). Focal Loss for Dense Object Detection. IEEE Trans. Pattern Anal. Mach. Intell..

[40] Zhang H., Xu C., Zhang S. (2023). Inner-IoU: More Effective Intersection over Union Loss with Auxiliary Bounding Box. arXiv.

[41] Tong Z., Chen Y., Xu Z., Yu R. (2023). Wise-IoU: Bounding Box Regression Loss with Dynamic Focusing Mechanism. arXiv.

[42] Hashemi S.R., Mohseni S., Erdogmus D., Prabhu S.P., Warfield S.K., Gholipour A. (2018). Tversky as a Loss Function for Highly Unbalanced Image Segmentation Using 3D Fully Convolutional Deep Networks.

[43] Bartlett P.L., Wegkamp M.H. (2008). Classification with a reject option using a hinge loss. J. Mach. Learn. Res..

[44] Chen L.-C., Zhu Y., Papandreou G., Schroff F., Adam H. (2018). Encoder-Decoder with Atrous Separable Convolution for Semantic Image Segmentation. arXiv.

[45] Badrinarayanan V., Kendall A., Cipolla R. (2017). SegNet: A Deep Convolutional Encoder-Decoder Architecture for Image Segmentation. IEEE Trans. Pattern Anal. Mach. Intell..

[46] Radford A., Kim J., Hallacy C., Ramesh A., Goh G., Agarwal S., Sastry G., Askell A., Mishkin P., Clark J. (2021). Learning Transferable Visual Models from Natural Language Supervision. arXiv.

[47] Huang S.-C., Shen L., Lungren M.P., Yeung S. GLoRIA: A Multimodal Global-Local Representation Learning Framework for Label-Efficient Medical Image Recognition. Proceedings of the 2021 IEEE/CVF International Conference on Computer Vision (ICCV).

